# ENHANCING PATIENT SATISFACTION IN EMERGENCY MEDICINE: A QUALITY IMPROVEMENT INITIATIVE TO IMPROVE PRESS GANEY SCORES THROUGH PHYSICIAN EDUCATION AND COMMUNICATION STRATEGIES

**DOI:** 10.51894/001c.122916

**Published:** 2024-08-31

**Authors:** Ayman Alhadheri

**Affiliations:** 1 Emergency Medicine McLaren Oakland

## 46

### INTRODUCTION

In healthcare quality measurement, patient satisfaction is increasingly acknowledged as a vital component and critical indicator. In the US, Press Ganey scores are commonly employed for this assessment. Our hospital’s Emergency Department (ED) faced a challenge with its lower-than-expected Press Ganey scores which necessitated an intervention for further enhancement and improvement.

### OBJECTIVES

This study explored the impact of a Quality Improvement (QI) project aimed at increasing awareness of the Press Ganey grading criteria amongst ED Physicians and improving Press Ganey scores in the ED using a Plan-Do-Study-Act (PDSA) cycle, thus enhancing patient satisfaction. Applicable skills emphasized in multiple methods.

### METHODS

This non-human subject research QI project, approved by the Institutional Review Board, employed a Plan-Do-Study-Act (PDSA) cycle approach. The intervention consisted of an educational workshop for ED Physicians, focusing on the nuances of Press Ganey grading and effective patient care strategies. The workshop included interactive elements such as role-playing and video presentations to simulate real-life scenarios. Physicians were taught specific skills, including how to effectively introduce themselves, communicate diagnostic and treatment plans, and acknowledge patient wait times. Press Ganey scores were collected and analyzed both before and after the intervention.

### RESULTS

The statistical analysis of the collected data revealed a significant improvement in Press Ganey scores following the educational intervention. The goal set by the ED medical director was met on all questions. There was one overall category increase of 8.13 in the average satisfaction score. Based on Chi-square test results, there was a statistically significant finding notable in the scores relating to Physician-patient communication and the clarity of care explanations provided to patients. This improvement suggests a direct correlation between the educational intervention and increased patient satisfaction.

### CONCLUSION

The increase in Press Ganey scores post-intervention illustrates the effectiveness of targeted educational programs in enhancing patient experience in the ED. Strengths of the study include its practical application and immediate impact. Limitations of the study include its focus on a single center and lack of long-term follow-up data. Future research should aim to explore the long-term effects of such interventions and their applicability in diverse healthcare settings.

